# Nicotine Addiction and Intensity of e-Cigarette Use by Adolescents in the US, 2014 to 2021

**DOI:** 10.1001/jamanetworkopen.2022.40671

**Published:** 2022-11-07

**Authors:** Stanton Glantz, Abra Jeffers, Jonathan P. Winickoff

**Affiliations:** 1 Retired, San Francisco, California; 2 Division of General Academic Pediatrics, Massachusetts General Hospital for Children, Boston; 3 Tobacco Research and Treatment Center, Massachusetts General Hospital, Boston; 4 Julius B. Richmond Center, American Academy of Pediatrics, Itasca, Illinois

## Abstract

**Importance:**

As e-cigarettes have become more effective at delivering the addictive drug nicotine, they have become the dominant form of tobacco use by US adolescents.

**Objective:**

To measure intensity of use of e-cigarettes, cigarettes, and other tobacco products among US adolescents and their dependence level over time.

**Design, Setting, and Participants:**

This survey study analyzed the cross-sectional National Youth Tobacco Surveys from 2014 to 2021. Confirmatory analysis was conducted using Youth Behavioral Risk Factor Surveillance System from 2015 to 2019. The surveys were administered to national probability samples of US students in grades 6 to 12.

**Exposures:**

Use of e-cigarettes and other tobacco products before and after the introduction of e-cigarettes delivering high levels of nicotine.

**Main Outcomes and Measures:**

First tobacco product used, age at initiation of use, intensity of use (days per month), and nicotine addiction (measured as time after waking to first use of any tobacco product).

**Results:**

A total of 151 573 respondents were included in the analysis (51.1% male and 48.9% female; mean [SEM] age, 14.57 [0.03] years). Prevalence of e-cigarette use peaked in 2019 and then declined. Between 2014 and 2021, the age at initiation of e-cigarette use decreased, and intensity of use and addiction increased. By 2017, e-cigarettes became the most common first product used (77.0%). Age at initiation of use did not change for cigarettes or other tobacco products, and changes in intensity of use were minimal. By 2019, more e-cigarette users were using their first tobacco product within 5 minutes of waking than for cigarettes and all other products combined. Median e-cigarette use also increased from 3 to 5 d/mo in 2014 to 2018 to 6 to 9 d/mo in 2019 to 2020 and 10 to 19 d/mo in 2021.

**Conclusions and Relevance:**

The changes detected in this survey study may reflect the higher levels of nicotine delivery and addiction liability of modern e-cigarettes that use protonated nicotine to make nicotine easier to inhale. The increasing intensity of use of modern e-cigarettes highlights the clinical need to address youth addiction to these new high-nicotine products over the course of many clinical encounters. In addition, stronger regulation, including comprehensive bans on the sale of flavored tobacco products, should be implemented.

## Key Points

**Question:**

How are e-cigarettes associated with nicotine addiction among US adolescents?

**Findings:**

In this survey study of 151 573 respondents, age at initiation of e-cigarette use decreased and intensity of use and addiction increased between 2014 and 2021. By 2019, more e-cigarette users were using their first tobacco product within 5 minutes of waking than users of cigarettes and all other tobacco products combined.

**Meaning:**

These findings suggest that clinicians need to be ready to address youth addiction to these new highly addictive nicotine products during many clinical encounters, and stronger regulation is needed, including comprehensive bans on the sale of flavored tobacco products.

## Teaser

This survey study assesses changes in measures of dependence and intensity of e-cigarette use among US adolescents on an annual basis between 2014 and 2021.

## Introduction

Electronic cigarettes (e-cigarettes) are highly engineered drug delivery devices that create and sustain addiction. Early e-cigarettes did not deliver nicotine as efficiently as cigarettes because they delivered freebase nicotine that was hard to inhale. This situation changed with the 2015 introduction of Juul products (Juul Labs Inc),^1^ which added benzoic acid to the nicotine e-liquid to lower the pH level and form protonated nicotine. Protonated nicotine increases addictive potential by making it easier to inhale quantities of nicotine that are difficult for naive users to achieve with cigarettes or earlier e-cigarettes.^2^ By 2018, Juul held 75% of the market.^3^ After the US Food and Drug Administration partially banned cartridge-based flavored products in 2020,^4^ disposable flavored protonated nicotine e-cigarettes rapidly gained adolescent market share^3,5^; in 2021 middle and high school students used Puff Bar (Puff Bar [26.8%]), Vuse (R. J. Reynolds Vapor Company [10.5%]), SMOK (Shenzhen IVPS Technology Co Ltd [8.6%]), and Juul (6.8%).^6^

In the brain, nicotine attaches to acetylcholine receptors and releases dopamine, which causes feelings of pleasure,^7,8,9^ upregulates acetylcholine receptors,^10^ and alters brain circuitry involved in learning, stress, and self-control, resulting in addiction and dependence.^11,12,13^ Adolescents and young adults are particularly susceptible to nicotine receptor upregulation and addiction because of enhanced brain plasticity.^14,15,16^

Prior studies reported changing prevalence of e-cigarette use among middle and high school students.^6,17,18,19,20,21,22,23,24^ By 2019, the Centers for Disease Control and Prevention (CDC) National Youth Tobacco Survey (NYTS) estimated that 5.3 million middle and high school students were using e-cigarettes.^22^ This number dropped to 3.6 million in 2020^23^ and again to 2.1 million in 2021 during the COVID-19 pandemic.^6^

One prior study^25^ assessed changes in nicotine dependence after the introduction of Juul. Population Assessment of Tobacco and Health data for individuals aged 12 to 34 years from 2014 to 2016 and 2017 to 2019 revealed increased daily use and nicotine dependence among adolescents aged 14 to 17 years from 2017 to 2019, after the introduction of Juul, with no change in the 2014 to 2016 cohort. Another study^26^ using NYTS data from 2015 to 2018 showed that high-frequency use of e-cigarettes and cigarettes was associated with higher odds of nicotine dependence (using tobacco products within 5 minutes of waking). Neither study tracked intensity of e-cigarette use, age at initiation, or dependence over time. This study moves beyond population prevalence as a measure of the changing e-cigarette use patterns among US adolescents over time to capture changes in measures of dependence and intensity of e-cigarette use among adolescents on an annual basis, during an 8-year period from 2014 through 2021.

## Methods

### Data

In this survey study, we analyzed data from the NYTS, a nationally representative survey of middle and high school students.^27^ We used 2014 through 2021, all years in which the NYTS provided information on number of days per month that respondents used e-cigarettes, cigarettes, cigars (including cigarillos and little cigars), and smokeless tobacco (ie, chewing tobacco, snuff, or dip). This was a secondary analysis of 2 deidentified public use data sets released by the CDC and was therefore deemed exempt from human participant research per the Massachusetts General Brigham Human Research Committee. This study followed the American Association for Public Opinion Research (AAPOR) reporting guideline.

Because of COVID-19, the 2021 NYTS transitioned from an in-person, tablet-based administration to a fully online administration where students could participate in classrooms, at home, or in another remote learning environment. Because of these differences in data collection, the CDC recommended that 2021 NYTS not be compared with earlier years.^6^ Indeed, the NYTS 2021 data demonstrate higher prevalence of e-cigarette use in those who took the survey at school (15.0%) vs at home (8.1%).^6^ To clarify behavioral reporting in different learning environments, the CDC performed the Adolescent Behaviors and Experiences Survey between January and June 2021, reporting current e-cigarette use rates of 25.2% among in-person, 17.2% among hybrid, and 9.1% among home-based high school students.^28^ These results are consistent with data from the 1990s and early 2000s showing that in-home surveys report lower prevalence of smoking than in-school surveys.^29,30,31^ Nevertheless, with these cautions and caveats in mind, and because this study focused on changes in consumption patterns within self-reported ever and current users of tobacco products, we have included the 2021 data for these users in our analysis. We reasoned that students who reported that they use tobacco products are less likely to be concerned about reporting details of their use patterns.

eTable 1 in the Supplement lists all the specific questions and variable definitions used in the analysis. *Ever use* of a tobacco product was coded as “yes” if the respondent reported ever using the product, even 1 or 2 times. *Current use* was coded as “yes” if the respondent reported using the product 1 or more of the past 30 days. There was no required threshold of lifetime use (such as 100 cigarettes in their lifetime) for current use. *Dual use* was coded “yes” if respondents reported current use of 2 or more tobacco products in the same survey year. We used the CDC’s categorization of days used for each product in the past 30 days established in 2014: 0, 1 to 2, 3 to 5, 6 to 9, 10 to 19, 20 to 29, and 30. The days used for each product among current users are tabulated separately, so each one includes users of that product as well as dual users and polyusers of other products.

We determined first tobacco product used with the question, “How old were you when you first smoked a cigarette, even 1 or 2 puffs?” and equivalent questions for other products. Respondents who gave the same age for multiple products were treated as having started using those products in the same year.

We assessed respondents’ level of tobacco dependence using the standard question^32^: “How soon after you wake up do you want to use a tobacco product of any kind?” The responses were coded as 0 for “I do not want to use tobacco products”; 1 for “I rarely want to use tobacco products”; 2 for “after more than 1 hour but less than 24 hours”; 3 for “from more than 30 minutes to 1 hour”; 4 for “from 6 to 30 minutes”; and 5 for “within 5 minutes.” The time to first cigarette is a commonly used measure of nicotine dependence in tobacco control research because it is a strong and consistent factor associated with smoking cessation success.^33^

We repeated our primary analyses with the CDC Youth Behavioral Risk Surveillance System^34^ (YRBSS) data, a nationally representative in-school survey of high school students. We used 2015, 2017, and 2019, all years in which the YRBSS provided information on the number of days per month that respondents used e-cigarettes, cigarettes, cigars, and smokeless tobacco. Details of the YRBSS analysis are provided in the eMethods in the Supplement.

### Statistical Analysis

We used CDC-provided weights and stratification variables to adjust for clustering and nonresponse and to match sample characteristics to national estimates. Results were tabulated for each year accounting for the complex survey design and weights for each year using Stata, version 15.0 (StataCorp LLC), commands syv, subpop():proportion, svy, subpop():regress, and svy, subpop():logistic. To test for trends over time while accounting for the uncertainties in each year’s estimates, metaregressions^35^ were computed for trends in proportion of adolescents in each intensity of use group (days per month) across years based on point estimates and SEs for each year using the Stata command *metareg*.

## Results

A total of 151 573 respondents were included in the analysis (mean [SEM] age, 14.57 [0.03] years). Because all respondents were in middle or high school, 99% of respondents were between 11 and 18 years of age, with 86% between 12 and 17 years of age. A total of 51.1% of the sample were male and 48.9% were female (weighted numbers).

**Figure 1.**
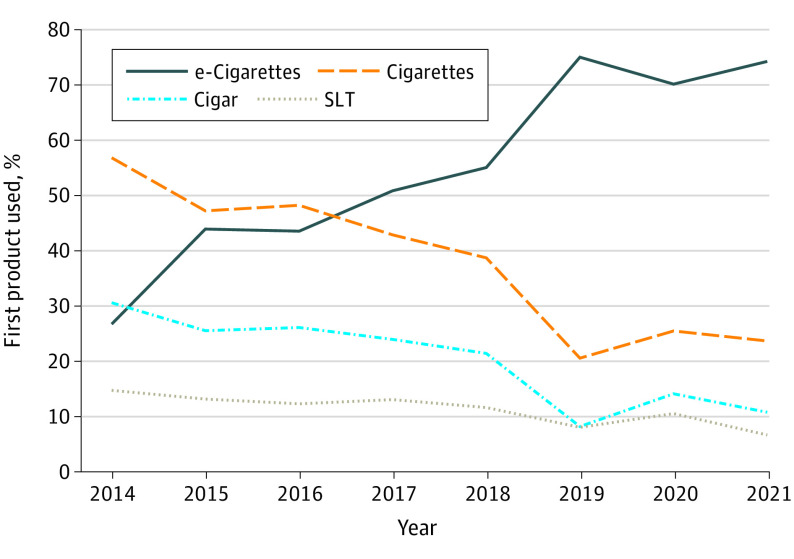
First Tobacco Product Used Among adolescents who were currently using any tobacco product, the proportion who initiated use with e-cigarettes increased over time, becoming the dominant first product in 2017. Percentages do not add up to 100% because some users initiated with more than 1 tobacco product at the same age. SLT indicates smokeless tobacco.

Among adolescents who currently use any tobacco product, the proportion whose first tobacco product used was e-cigarettes increased from 27.2% in 2014 to 78.3% in 2019 and remained at 77.0% in 2021 (Figure 1). By 2017, e-cigarettes were the most popular initial tobacco product. For each year from 2019 to 2021, more current tobacco users were initiating use with e-cigarettes than all other products combined (Figure 1 and eFigure 1 in the Supplement).

**Figure 2.**
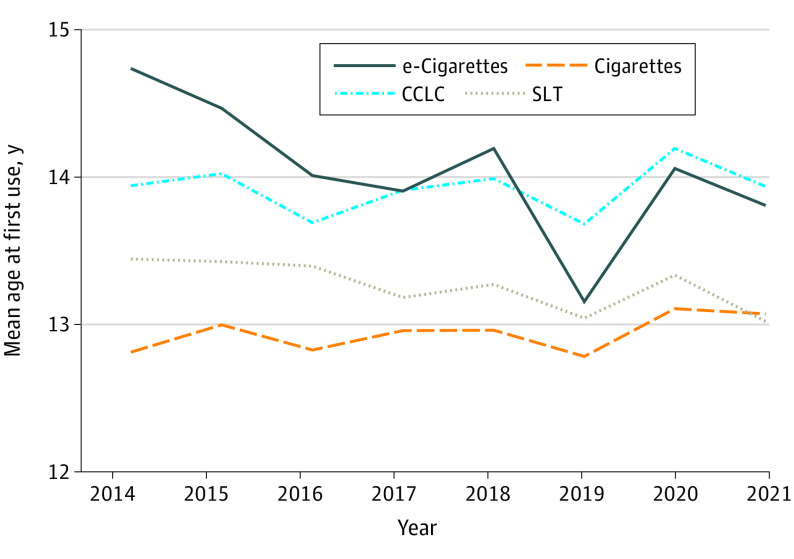
Mean Age at First Use of Tobacco Products Mean age at first use decreased over time for e-cigarettes but remained stable for other tobacco products. Regression analyses are shown in eTable 2 in the Supplement. CCLC indicates cigar, cigarillo, and little cigar; SLT, smokeless tobacco.

Age at first use for e-cigarettes decreased by −0.159 (95% CI, −0.176 to 0.143) years (1.9 months) per calendar year (*P* < .001), controlling for respondent age at the time they completed the survey (Figure 2 and eTable 2 in the Supplement). In contrast, change in age at first use for cigarettes (0.017 [95% CI, −0.011 to 0.045] years; *P* = .24), cigars (0.015 [95% CI, −0.011 to 0.041] years; *P* = .25), and smokeless tobacco (−0.036 [95% CI, −0.074 to 0.0002] years; *P* = .64) was not significant.

**Figure 3.**
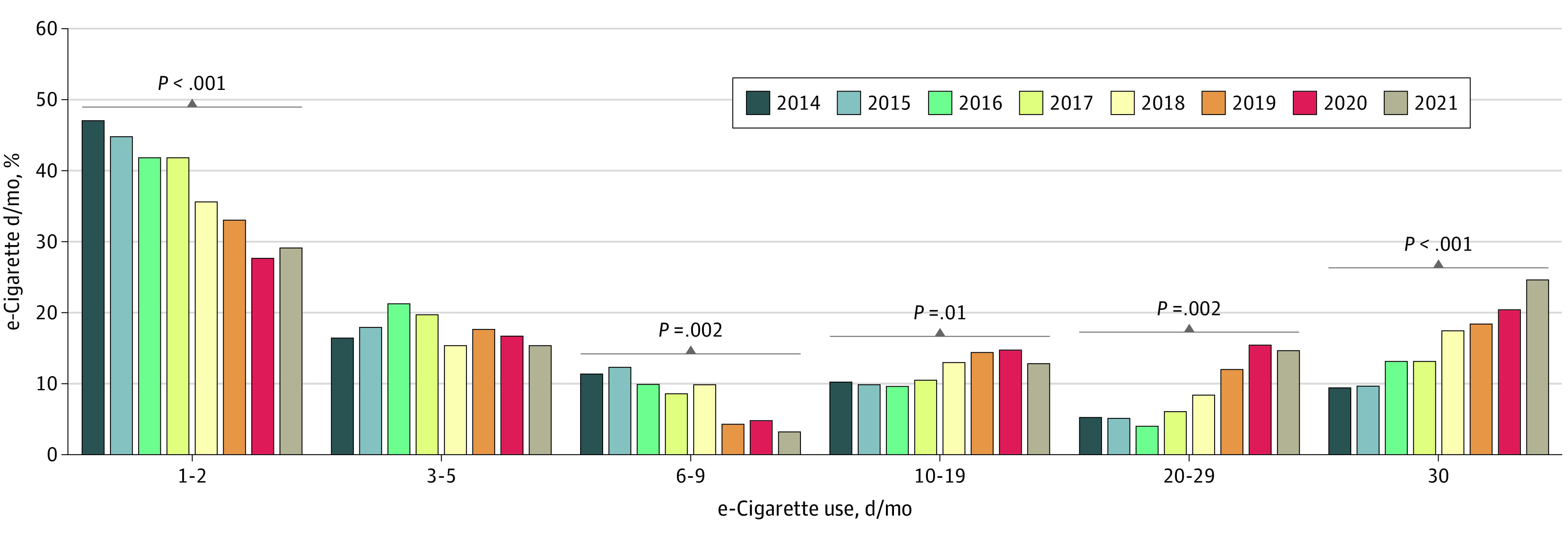
Days of e-Cigarette Use per Month by Adolescents Between 2014 and 2021, days of e-cigarette use per month shifted from light use to heavier use. Statistical results are shown in eTable 3 in the Supplement.

Intensity of e-cigarette use shifted from using 9 or fewer days a month to 10 or more days a month (Figure 3 and eTable 3 in the Supplement). This shift in e-cigarette use intensity is also reflected in median number of days used, which increased from 3 to 5 d/mo in 2014 to 2018 to 6 to 9 d/mo in 2019 to 2020 and 10 to 19 d/mo in 2021. Intensity of use of cigarettes, cigars, and smokeless tobacco generally did not shift over time (eFigure 3 and eTable 3 in the Supplement).

**Figure 4.**
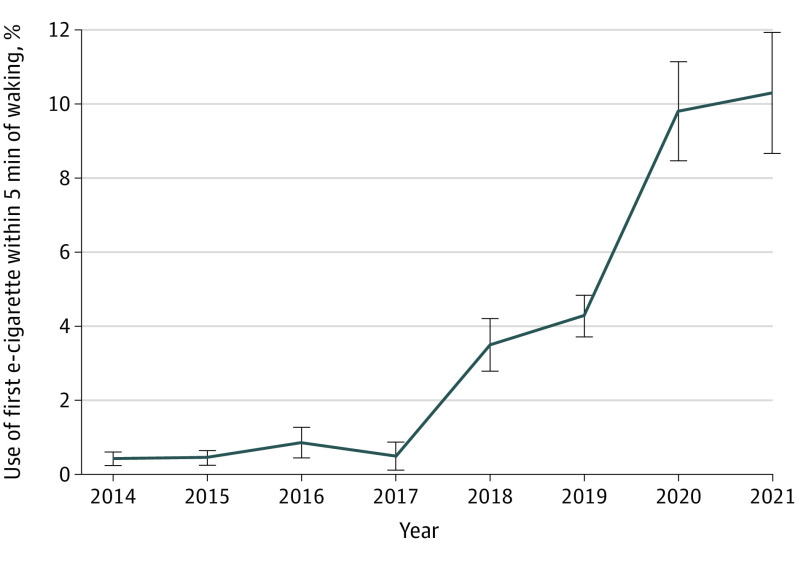
Use of e-Cigarettes Within 5 Minutes of Waking Includes e-cigarette users who used no other tobacco products. After remaining stable from 2014 to 2017 (*P* = .74 by interrupted time series analysis), the proportion of users who consumed their first e-cigarette within 5 minutes of waking rapidly increased more than 10-fold (*P* = .002 for slope change following 2017 by interrupted time series analysis). Error bars indicate SEs.

Addiction to e-cigarette nicotine, measured as the odds of having use of first tobacco product within 5 minutes of waking, increased over time for sole e-cigarette users (eTable 4 in the Supplement). These changes over time were not uniform. From 2014 to 2017, the percentage of sole e-cigarette users who used e-cigarettes within 5 minutes of waking was less than 1% (Figure 4 and eTable 4 in the Supplement). However, beginning in 2017 through 2021, a shift occurred, with 10.3% using their first e-cigarette within 5 minutes of waking by 2021. During this same time, addiction did not change for sole cigarette smokers (odds ratio [OR] per year, 0.99 [95% CI, 0.85-1.16]; *P* = .92) or smokeless tobacco users (OR per year, 0.97 [95% CI, 0.81-1.16]; *P* = .73), but did increase among sole cigar users (OR per year, 1.49 [95% CI, 1.16-1.90]; *P* = .002) (eTable 4 in the Supplement). For comparison, between 2014 and 2021, a mean (SE) of 6.1% (0.8%) of smokers with sole use of cigarettes first used cigarettes within 5 minutes of waking and 5.2% (1.0%) of smokeless tobacco users used the product within 5 minutes of waking. The highest mean (SE) percentage for sole cigar users was 6.3% (2.4%) in 2020.

**Figure 5.**
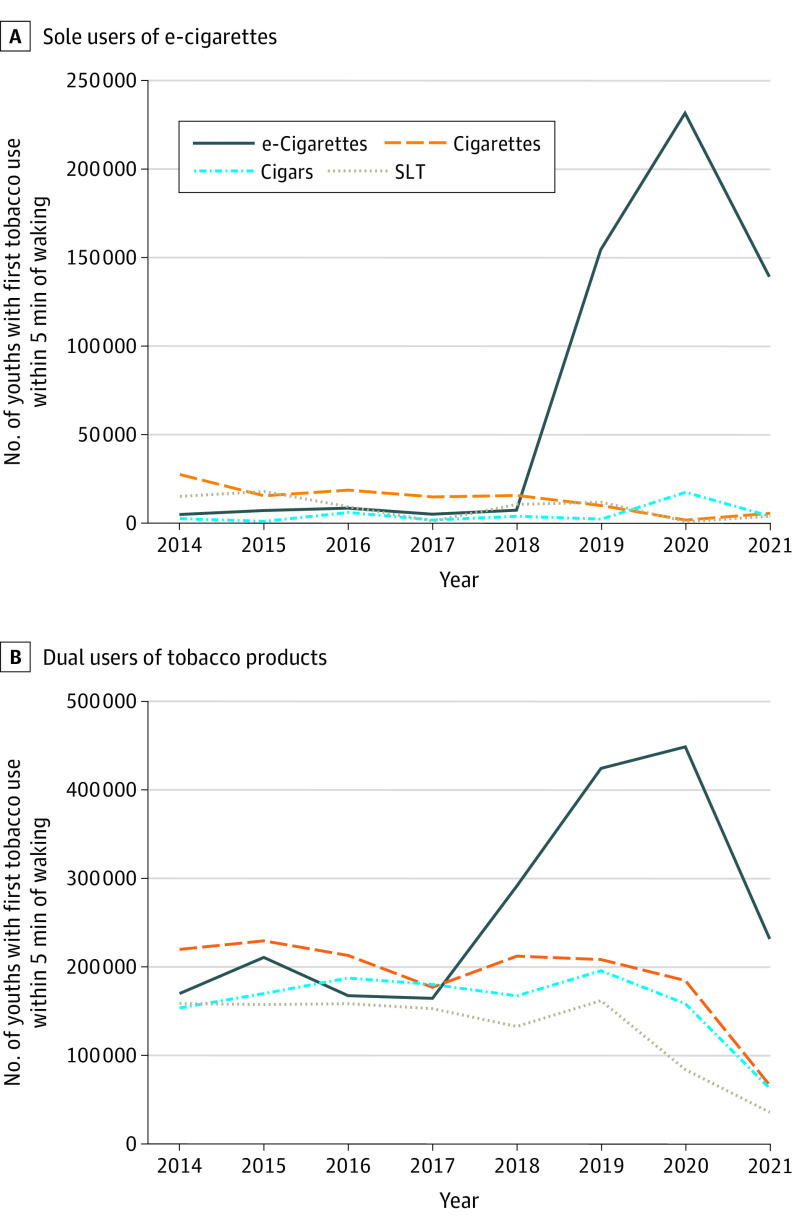
Use of Any Tobacco Products Within 5 Minutes of Waking A, The estimated number of adolescents with high levels of nicotine dependence and who were sole users of e-cigarettes diverged from the numbers for other products in 2018, exceeding the sum of use of cigarettes and all other products combined. B, Considering dual-product users with the other products confirms that e-cigarette users began to exceed use of all other tobacco products among adolescents with high levels of nicotine dependence. The 2021 numbers may underestimate tobacco use compared with earlier years, as discussed in the Limitations subsection of the Discussion section. SLT indicates smokeless tobacco.

To isolate the population effect of addictiveness of different product types, we considered those adolescents who used only 1 product type and measured use of the product within 5 minutes of waking (Figure 5). Beginning in 2019, the number of addicted e-cigarette users (n = 177 000) exceeded the numbers of all other tobacco product users (n = 23 000) on this high-addiction measure.

Results from the YRBSS were similar to those of the NYTS (eAppendix, eTable 5, and eFigures 4 and 5 in the Supplement), showing a shift in e-cigarette use to more days per month with minimal changes in patterns of cigarette use. The frequent use of e-cigarettes (≥20 days in the past month) increased between 2017 and 2019 to the point where it was 700% higher than frequent use of cigarettes by 2019.

## Discussion

In 2017, e-cigarettes became the most common first tobacco product used, with the proportion of adolescents who initiated tobacco product use with e-cigarettes increasing over time (Figure 1) and the age at initiation of e-cigarette use decreasing (Figure 2). In addition, measures of addiction increased: days per month (Figure 3) and the fraction of users who used their first product within 5 minutes of waking (Figure 4) increased, and e-cigarette addiction surpassed that for all other forms of tobacco products combined (Figure 5). Age at initiation of use did not change for cigarettes and other products, and changes in intensity of use for them were minimal.

As has been reported elsewhere,^6,17,18,19,20,21,22,23,24^ the historic decline in use of tobacco products by US adolescents reversed after the advent of e-cigarettes, with e-cigarette use peaking in 2019 at a higher level than cigarette smoking in 2006 (eFigure 2 in the Supplement). The shift to e-cigarettes being the first tobacco product used is consistent with Monitoring the Future data from 8th and 10th grade students in 2015 to 2017.^36,37^ Likewise, the decrease in age at initiation of e-cigarette use but not use of other tobacco products is consistent with earlier NYTS results^38^: the 8.8% of ever users of e-cigarettes aged 16 to 17 years who initiated use at 14 years or younger in 2014 increased to 28.6% in 2018, whereas initiation age did not change for cigarettes, cigars, and smokeless tobacco products. Our results are consistent with the prior Population Assessment of Tobacco and Health study that showed increased intensity of use and nicotine dependence among adolescents through 2019 after the introduction of Juul and the diffusion of protonated nicotine technology in e-cigarettes^25^; our results also show that the shift toward increased e-cigarette use and higher levels of addiction continued through 2021. In addition, the 2022 NYTS reported that 2.6 million adolescents used e-cigarettes and 27.6% of them used e-cigarettes daily; the comparable numbers reported herein for 2021 are 2.1 million and 24.7%.^39^ Our findings are also consistent with the finding that using e-cigarettes on more days per month is associated with higher levels of nicotine dependence.^26^

The decrease in e-cigarette use from 2019 levels may be attributable to a variety of factors, including local, state, and national strategies to address e-cigarette use among adolescents and enacting comprehensive restrictions on the sale of flavored tobacco products,^40,41,42^ as well as raising the federal minimum age for tobacco product sale to 21 years.^43^ Heightened health concerns because of e-cigarette– or vaping–associated lung injury^44^ may also have contributed to this decrease. The effects of COVID-19 on the 2021 results are likely more reflected in the prevalence estimates than in the measures of behavior among self-identified tobacco product users. The fact that adolescents were predominately at home and outside social environments may have affected tobacco use. A national online survey in May 2020, during the COVID-19 pandemic, found that 36.5% of adolescents and young adults (aged 13 to 20 years) who used e-cigarettes reported quitting and another 2.2% switched to other nicotine products.^45^

The extent to which decreases in tobacco product use for the 2021 NYTS data may reflect a long-term trend, owing perhaps to education about e-cigarettes, increased concern about the health risks of e-cigarettes,^44^ restrictions on available products such as the US Food and Drug Administration’s 2020 partial ban on flavored products,^4^ short-term lack of access to products and stimuli for use among peers, biases in the data collection, or some combination of these factors is unknown. Some of this decrease may be an artifact due to NYTS being administered at home as well as in school. Although adolescent prevalence of e-cigarette use appeared to decrease in the NYTS, data from the CDC’s Adolescent Behaviors and Experiences Survey from January to June 2021 suggested that the in-person high school student respondents in 2021 had rates of current e-cigarette use of 25.2% (95% CI, 13.9%-41.2%),^28^ similar to the 2019 NYTS levels of 27.5% (95% CI, 23.5%-29.7%)^22^ measured among in-person high school student respondents. It will be important to determine to what degree prevalence of e-cigarette use among adolescents rebounds as the return to school and contact with their peers and society generally proceeds.

Regardless of falling prevalence, e-cigarette users were initiating use at younger ages, use became more intensive (in days per month), and a higher percentage used them within 5 minutes of waking. These changes may reflect the increased addictive potential of protonated nicotine delivery products that make it easier to inhale nicotine than from cigarettes or other combustible tobacco products.^1,2^ The fact that e-cigarette addiction trends are continuing to increase despite the 2019 federal legislation raising the tobacco sales age to 21 years suggests that tighter regulation, additional legislative action, or both may be necessary to protect adolescents.

Despite the COVID-19 pandemic leading to people being socially isolated, students being out of school, and the increased risk of adolescents and young adults contracting COVID-19 with e-cigarette use,^46^ intensity of use among adolescents continued to increase. This increase in intensity may reflect increasing use of nicotine for self-medication in response to increases in adolescent depression, anxiety, tic disorders, and suicidality that occurred during the COVID-19 pandemic.^28^ The pandemic has also been a lost year for school-based prevention and treatment efforts, meaning that abatement plans will need to be intensified to address the nicotine addiction in those adolescents who missed a year of contact with adults who might have otherwise helped them get treatment.^47^

The change in e-cigarette use and intensity of use among adolescents contrasts with what was happening in the adult e-cigarette market at the same time.^48,49,50^ The prevalence of current adult e-cigarette use in the Tobacco Use Supplement to the Current Population Survey showed an upward trend from 2010 until 2014,^51^ followed by a decline to the 2019 rate of 2.3%,^52^ less than one-tenth of the 27.5% prevalence among high school respondents in the NYTS that year.^22^ High-dose concealable nicotine e-cigarettes entered the market as a product targeting adolescents, as shown by the fact that by 2018, only 2% of adults used a flash drive–shaped e-cigarette regularly.^53^ Despite several years of intensive adolescent e-cigarette users becoming 18 years of age and therefore part of a different category, the 2020 National Health Interview Survey found only 3.7% prevalence of adult e-cigarette use,^54^ compared with 19.6% among high school students.^23^ These findings suggest that the primary effect of the modern e-cigarette has been to addict adolescents to nicotine. Clinicians should be vigilant for new tobacco products that may come into the youth tobacco product market.

### Limitations

The primary limitation of this study is that it was based on the NYTS, which is a cross-sectional survey. The NYTS collects self-reported data from respondents without biochemical verification of tobacco use behavior, which could lead to recall bias.

In addition to the changes in how the NYTS was administered in 2021, the NYTS was conducted via paper-and-pencil questionnaires until 2019, when it shifted to electronic data collection. In 1 study,^30^ paper-administered questionnaires tended to result in nonsignificantly lower prevalence reporting. Beginning in 2019, the electronic survey contained skip patterns and tobacco product images, which may limit comparability with data collected via paper-and-pencil surveys, in which respondents were asked to answer all questions (regardless of tobacco product use) and did not have any images to aid with product recall. In addition, owing to COVID-19, 2020 NYTS data collection ended early, in March, yielding a smaller sample size and lower response rate than usual. The CDC performed additional nonresponse bias analysis assessing differences in responding and nonresponding schools for 2020 and concluded that they were able to create survey weights that compensated for these problems.^23^

## Conclusions

Use of e-cigarettes reversed the long-term decline in US youth tobacco use and expanded the tobacco epidemic by attracting many adolescents at low risk of initiating nicotine use with cigarettes.^55,56,57^ This survey study found that between 2014 and 2021, although prevalence of e-cigarette use among adolescents peaked in 2019 and then declined, the age of initiation among ever users continued to decrease and the intensity of use and level of addiction among adolescents who are current e-cigarette users increased. This increasing intensity of use may reflect the higher nicotine delivery and addiction liability of e-cigarettes that use protonated nicotine.^1,2^ Clinicians should question all their patients about nicotine and tobacco product use, including e-cigarettes and other new nicotine products. Because tobacco addiction is a chronic disease, clinicians should be ready to address youth addiction to these new high-nicotine products during the course of many clinical encounters. The increasing intensity of use of modern e-cigarettes highlights the need for local, state, and federal comprehensive bans on the sale of flavored tobacco products and consideration of ending the sale of these products on the open retail market, as has been done in 47 countries as of 2021.^58^

## Notes

Supplement. **eFigure 1.** Number of Adolescents Using Different Tobacco Products **eFigure 2.** Current e-Cigarette and Cigarette Use, Separating Out Dual Use (Reporting Current Use of Both e-Cigarettes and Cigarettes in the Same Survey) **eFigure 3.** Days per Month Adolescents Used Cigarette, Cigars, and Smokeless Tobacco **eTable 1.** NYTS Variables Used in the Analysis **eTable 2.** Age of Tobacco Product Initiation Among Ever Users **eTable 3.** Metaregressions for Changes in Tobacco Product Use Intensity Over Time, 2014-2021 **eTable 4.** Use Within 5 Minutes of Waking Among Sole Product Users **eMethods.** YRBSS Supplemental Analysis **eAppendix.** YRBSS Supplemental Analysis **eTable 5.** Current Tobacco Product Use in YRBSS and NYTS (High School Students) **eFigure 4.** e-Cigarette and Cigarette Use in YRBSS **eFigure 5.** Percentage of e-Cigarette and Cigarette Users Who Used Product ≥20 d/mo
