# Supplementary material for: Nicotine Addiction and Intensity of e-Cigarette Use by Adolescents in the US, 2014 to 2021
Source: JAMA Netw Open. 2022 Nov 7;5(11):e2240671. doi: 10.1001/jamanetworkopen.2022.40671 (PMC9641541; doi:10.1001/jamanetworkopen.2022.40671)
Supplement: Supplement. — eFigure 1. Number of Adolescents Using Different Tobacco Products eFigure 2. Current e-Cigarette and Cigarette Use, Separating Out Dual Use (Reporting Current Use of Both e-Cigarettes and Cigarettes in the Same Survey) eFigure 3. Days per Month Adolescents Used Cigarette, Cigars, and Smokeless Tobacco eTable 1. NYTS Variables Used in the Analysis eTable 2. Age of Tobacco Product Initiation Among Ever Users eTable 3. Metaregressions for Changes in Tobacco Product Use Intensity Over Time, 2014-2021 eTable 4. Use Within 5 Minutes of Waking Among Sole Product Users eMethods. YRBSS Supplemental Analysis eAppendix. YRBSS Supplemental Analysis eTable 5. Current Tobacco Product Use in YRBSS and NYTS (High School Students) eFigure 4. e-Cigarette and Cigarette Use in YRBSS eFigure 5. Percentage of e-Cigarette and Cigarette Users Who Used Product ≥20 d/mo [file jamanetwopen-e2240671-s001.pdf]

Supplementary Online Content

Glantz S, Jeffers A, Winickoff JP. Nicotine addiction and intensity of e-cigarette use by adolescents in the US, 2014 to 2021. *JAMA Netw Open*. 2022;5(11):e2240671. doi:10.1001/jamanetworkopen.2022.40671

eFigure 1. Number of Adolescents Using Different Tobacco Products
eFigure 2. Current e-Cigarette and Cigarette Use, Separating Out Dual Use (Reporting Current Use of Both e-Cigarettes and Cigarettes in the Same Survey)
eFigure 3. Days per Month Adolescents Used Cigarette, Cigars, and Smokeless Tobacco
eTable 1. NYTS Variables Used in the Analysis
eTable 2. Age of Tobacco Product Initiation Among Ever Users
eTable 3. Metaregressions for Changes in Tobacco Product Use Intensity Over Time, 2014-2021
eTable 4. Use Within 5 Minutes of Waking Among Sole Product Users
eMethods. YRBSS Supplemental Analysis
eAppendix. YRBSS Supplemental Analysis
eTable 5. Current Tobacco Product Use in YRBSS and NYTS (High School Students)
eFigure 4. e-Cigarette and Cigarette Use in YRBSS
eFigure 5. Percentage of e-Cigarette and Cigarette Users Who Used Product ≥20 d/mo

This supplementary material has been provided by the authors to give readers additional information about their work.

© 2022 Glantz S et al. *JAMA Network Open*.

eFigure 1. Number of Adolescents Using Different Tobacco Products

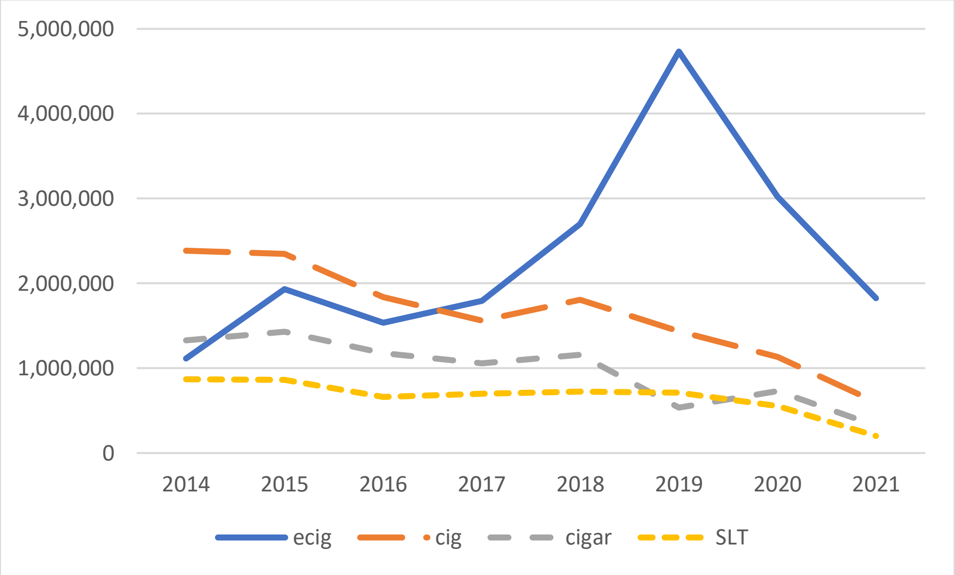

E-cigarettes became the most used first tobacco product in 2017, being used by more than all other first products combined beginning in 2019. Many students started with more than one product in the same year. As discussed in the main text, data from 2021 may underestimate tobacco use levels because of changes in the way the survey was administered, so the drop between 2020 and 2021 may be less than appears in this figure.

© 2022 Glantz S et al. *JAMA Network Open*.

eFigure 2. Current e-Cigarette and Cigarette Use, Separating Out Dual Use (Reporting Current Use of Both e-Cigarettes and Cigarettes in the Same Survey)

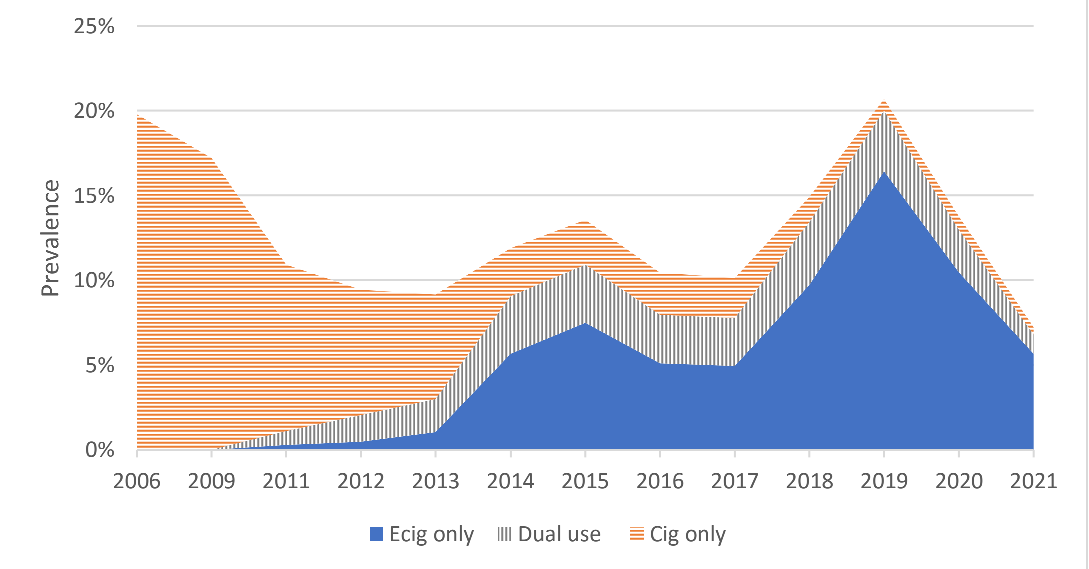

E-cigarette use started to substantially increase in 2014, when more youth were using e-cigarettes than cigarettes, with most cigarette smokers dual using with e-cigarettes. There was a dip in e-cigarette use from 2015 to 2017, possibly reflecting public health campaigns targeting e-cigarettes before the more addictive Juul product started to dominate the market. The historic decline in total cigarette plus e-cigarette use reversed after 2013 and peaked in 2019 at a higher level than cigarette smoking in 2006. Even with the drop in e-cigarette and cigarette use after 2019, prevalence of total use was at about the level last observed in 2013. E-cigarette use was not reported in NYTS in 2006 and 2009,[18] but use was not yet widespread; these years are included to provide long-term picture of changing use. Results for 2006 and 2009 are from CDC; years 2011 on were calculated by the authors. We did not include cigar and smokeless tobacco in this figure because doing so would generate many categories of dual and polyuse. As discussed in main text, the 2021 data may underestimate tobacco use levels because of changes in the way the survey was administered, so the drop between 2020 and 2021 may be less than appears in this figure.

© 2022 Glantz S et al. *JAMA Network Open*.

eFigure 3. Days per Month Adolescents Used Cigarette, Cigars, and Smokeless Tobacco

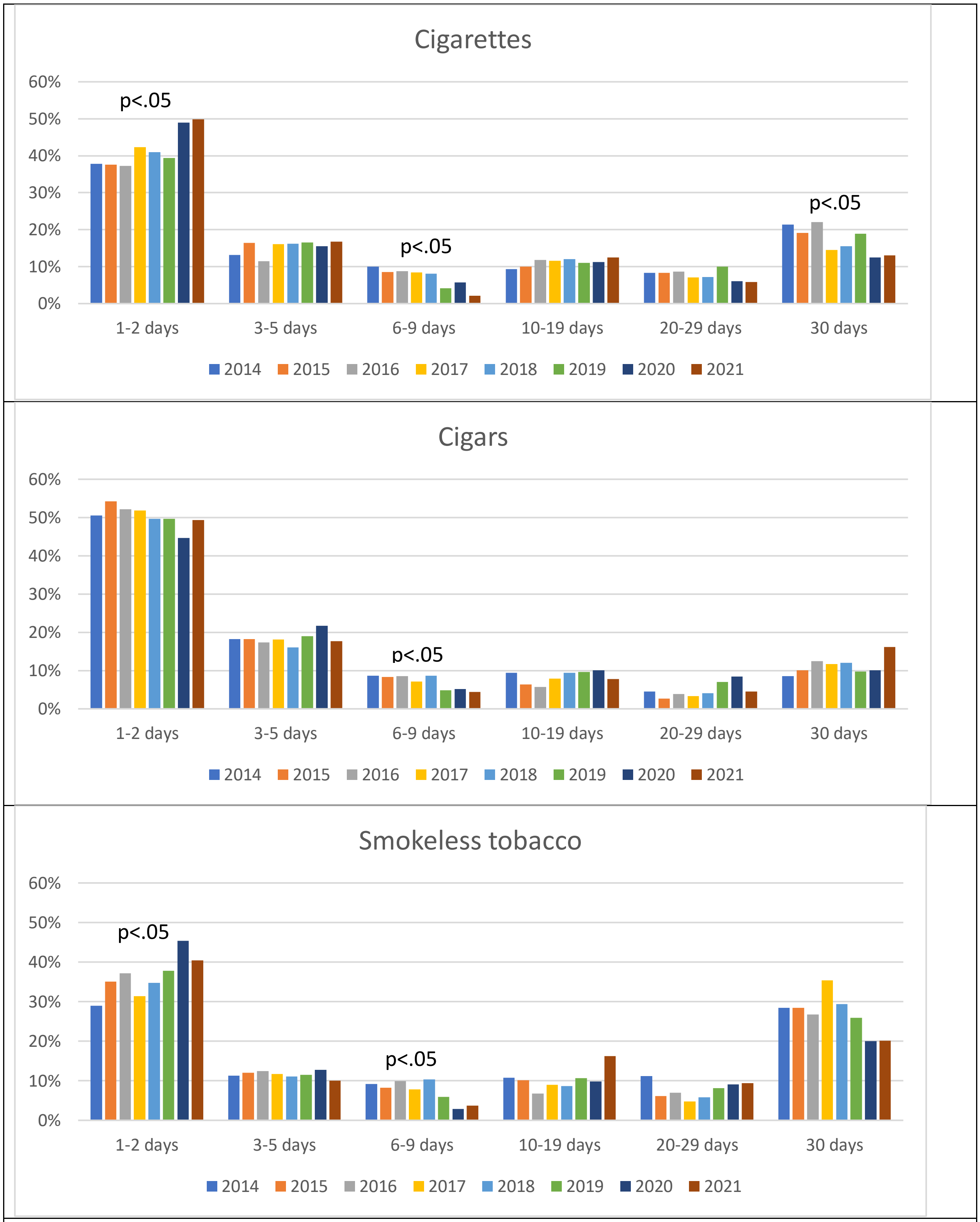

Consumption generally did not change significantly over time. (Exact p values in eTable 3.)

© 2022 Glantz S et al. *JAMA Network Open*.

**eTable 1.** NYTS Variables Used in the Analysis								
Question (on 2021 NYTS)	Year							
	2014	2015	2016	2017	2018	2019	2020	2021
How old are you?	qn1	qn1	Q1	Q1	Q1	Q1	Q1	QN1
What is your sex?	qn2	qn2	Q2	Q2	Q2	Q2	Q2	QN2
Recoded from:<br>Are you Hispanic, Latino, Latina, or of Spanish origin?<br>What race or races do you consider yourself to be? (Select one or more)	qn4<br>qn5	qn4<br>qn5	Q4<br>Q5	Q4<br>Q5	Q4<br>Q5	Q4<br>Q5	Q4<br>Q5	QN4A<br>QN5A-E
Have you ever smoked a cigarette, even one or two puffs?	qn7	qn6	Q7	Q7	Q7	Q6	Q22	QN35
How old were you when you first smoked a cigarette, even one or two puffs?	qn11	qn10	Q11	Q8	Q8	Q7	Q23	QN36
During the past 30 days, on how many days did you smoke cigarettes?	qn13	qn12	Q13	Q11	Q11	Q9	Q25	QN38
Have you ever used an e-cigarette, even once or twice?	qn31	qn28	Q26	Q28	Q28	Q34	Q6	QN6
How old were you when you first used an e-cigarette, even once or twice?	qn34	qn31	Q30	Q29	Q29	Q35	Q7	QN7
During the past 30 days, on how many days did you use e-cigarettes?	qn35	qn32	Q31	Q31	Q31	Q37	Q9	QN9
Have you ever smoked a cigar, cigarillo, or little cigar, even one or two puffs?	qn20	qn18	Q18	Q19	Q19	Q18	Q35	QN51
How old were you when you first smoked a cigar, cigarillo, or little cigar, even one or two puffs?	qn23	qn21	Q19	Q20	Q20	Q19	Q36	QN52
During the past 30 days, on how many days did you smoke cigars, cigarillos, or little cigars?	qn24	qn22	Q20	Q21	Q21	Q20	Q37	QN53
Have you ever used chewing tobacco, snuff, or dip, even just a small amount?	qn27	qn24	Q22	Q24	Q24	Q27	Q44	QN62
How old were you when you first used chewing tobacco, snuff, or dip, even just a small amount?	qn28	qn25	Q23	Q25	Q25	Q28	Q45	QN63
During the past 30 days, on how many days did you use chewing tobacco, snuff, or dip?	qn29	qn26	Q24	Q26	Q26	Q29	Q46	QN64
How soon after you wake up do you want to use a tobacco product of any kind?	qn46	qn48	Q56	Q52	Q52	Q64	Q62	QN91
First product used (among youth who used both cigarettes and e-cigarettes at least once)<br>Recode: Ecigarette first: ecig_first=1 if start_diff>0; Started both products in the same year: ecig_first=0 if start_diff==0; Cigarettes first: ecig_first=-1 if start_diff<0								

© 2022 Glantz S et al. *JAMA Network Open*.

**eTable 2.** Age of Tobacco Product Initiation Among Ever Users					
Age (95% CI)	Year of survey*	Age at time of survey† (years)	Constant	R^2	No.
e-cigarette	-0.159 (-0.176-0.143) p<.001	0.824 (0.810-0.838) p<.001	13.7 (13.6-13.7)	0.51	35,507
cigarette	0.017 (-0.011-0.045) p=.235	0.668 (0.642-0.695) p<.001	12.1 (12.0-12.2)	0.21	25,171
cigars	0.015 (-0.011-0.041) p=.253	0.775 (0.749-0.800) p<.001	12.8 (12.7-12.8)	0.31	18,618
smokeless tobacco	-0.036 (-0.074-0.002) p=.064	0.686 (0.650-0.722) p<.001	12.6 (12.4-12.7)	0.24	9,584
*Survey year, centered on 2014, the year of the first survey in the analytical sample<br>†Age centered on 14.4868, the mean age of all respondents.					

© 2022 Glantz S et al. *JAMA Network Open*.

eTable 3. Metaregressions for Changes in Tobacco Product Use Intensity Over Time, 2014-2021

Days/month	E-cigarettes				Cigarettes				Cigars (including cigarillos and little cigars)				Smokeless tobacco products (chewing tobacco, snuff, or dip)			
	slope	95%CI		p	Slope	95%CI		p	slope	95%CI		p	slope	95%CI		p
1-2 days	***-0.030***	***-0.036***	***-0.023***	***<0.001***	**0.016**	**0.005**	**0.027**	**0.013**	-0.006	-0.016	0.004	0.173	**0.017**	**0.004**	**0.031**	**0.020**
3-5 days	-0.003	-0.010	0.005	0.395	0.005	-0.004	0.013	0.227	0.001	-0.006	0.008	0.700	-0.001	-0.007	0.006	0.808
6-9 days	***-0.013***	***-0.019***	***-0.007***	***0.002***	***-0.011***	***-0.016***	***-0.006***	***0.002***	***-0.007***	***-0.012***	***-0.002***	***0.015***	***-0.009***	***-0.016***	***-0.003***	***0.012***
10-19 days	0.007	0.002	0.012	0.010	0.004	-0.001	0.009	0.117	0.003	-0.003	0.010	0.286	0.003	-0.006	0.012	0.493
20-29 days	**0.017**	**0.009**	**0.024**	**0.002**	-0.003	-0.008	0.002	0.254	0.004	-0.002	0.010	0.142	0.000	-0.009	0.010	0.980
30 days	**0.021**	**0.017**	**0.026**	**<0.001**	***-0.013***	***-0.022***	***-0.003***	***0.017***	0.005	-0.002	0.011	0.149	-0.012	-0.026	0.002	0.082

Bold italic = significantly falling over time. **Bold** = significantly increasing over time.

eTable 4. Use Within 5 Minutes of Waking Among Sole Product Users

Mean (SE)	E-cigarette		Cigarette		Cigar		Smokeless tobacco	
2014	0.4%	(0.2%)	7.2%	(1.8%)	0.4%	(0.3%)	5.6%	(2.1%)
2015	0.5%	(0.2%)	4.3%	(1.4%)	0.0%	-	7.2%	(3.1%)
2016	0.8%	(0.4%)	6.1%	(2.1%)	1.3%	(0.7%)	4.3%	(2.8%)
2017	0.5%	(0.4%)	5.0%	(1.9%)	0.2%	(0.2%)	0.5%	(0.5%)
2018	3.5%	(0.7%)	8.6%	(2.9%)	1.0%	(0.6%)	6.6%	(2.3%)
2019	4.3%	(0.6%)	9.1%	(3.9%)	0.4%	(0.4%)	8.0%	(3.8%)
2020	9.8%	(1.3%)	1.4%	(1.0%)	6.3%	(2.4%)	0.5%	(0.5%)
2021	10.3%	(1.6%)	6.3%	(5.3%)	2.3%	(1.6%)	6.5%	(3.5%)
Trend* (OR/yr) (95%CI)	1.687 (1.534-1.854)		0.992 (0.849-1.159)		1.486 (1.160-1.903)		0.969 (0.809-1.160)	
p	<0.001		0.920		0.002		0.730	
n†	9,124		1,332		1,788		869	

* Logistic regression against survey year (centered on 2014)
† Subpopulation size. Full sample sizes: 152,528, 152,388, 152,386,152,403

© 2022 Glantz S et al. *JAMA Network Open*.

eMethods. YRBSS Supplemental Analysis

We used the Centers for Disease Control and Prevention Youth Behavioral Risk Surveillance System[15] (YRBSS), a biannual nationally representative in-school survey of high school students from 2015, 2017, and 2019, all years in which the YRBS provided information on the number of days per month that respondents used e-cigarettes, cigarettes, cigars, and SLT.

Following the main NYTS analysis, “ever use” of a product was coded as “yes” if the respondent reported ever using the product, even 1 or 2 times. “Current use” was coded as “yes” if the respondent reported using the product 1 or more of the past 30 days. There was no required threshold of lifetime use (such as 100 cigarettes in their lifetime) for current use. “Dual use” contains respondents who report current use of both e-cigarettes and cigarettes in the same survey year.

eAppendix. YRBSS Supplemental Analysis

The YRBSS and NYTS results for high school students showed similar changes among the three years available for YRBSS, with the YRBSS generally yielding higher estimates of e-cigarette and cigarette (eTable 5).

eTable 5. Current Tobacco Product Use in YRBSS and NYTS (High School Students)

Year	E-cigarette only		Dual use		Cigarette only		Total e-cigarette		Total cigarette	
	YRBSS	NTYS	YRBS	NYTS	YRBS	NYTS	YRBS	NYTS	YRBS	NYTS
2015	15.5%	10.6%	7.2%	5.0%	3.1%	4.0%	22.7%	15.6%	10.3%	9.0%
2017	6.3%	7.3%	5.6%	4.0%	2.4%	3.6%	11.9%	11.3%	8.0%	7.6%
2019	26.6%	22.5%	4.8%	5.0%	0.6%	0.8%	31.4%	27.5%	5.4%	5.8%

Consistent with the NYTS results, the YRBSS showed a shift to more days of use among e-cigarette users, with little or no change over the same period of time for cigarettes, cigars and SLT (compare eFigure 4 with Figure 3 and eFigure 3). When limiting the NYTS data to high school students the same general pattern was present for e-cigarette and cigarettes as in the NYTS. Between 2015 and 2019, the percentage of e-cigarette users that used 20+ days per month shifted from 12% to 33%, while the percentage of cigarette users that used 20+ days per month shifted from 31% to 22% in YRBSS (eFigure 5).

© 2022 Glantz S et al. *JAMA Network Open*.

eFigure 4. e-Cigarette and Cigarette Use in YRBSS

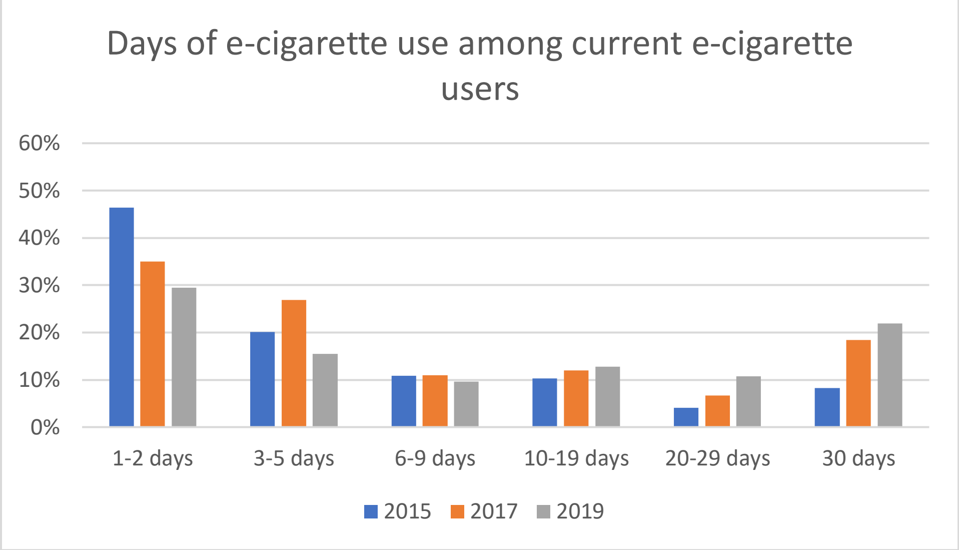

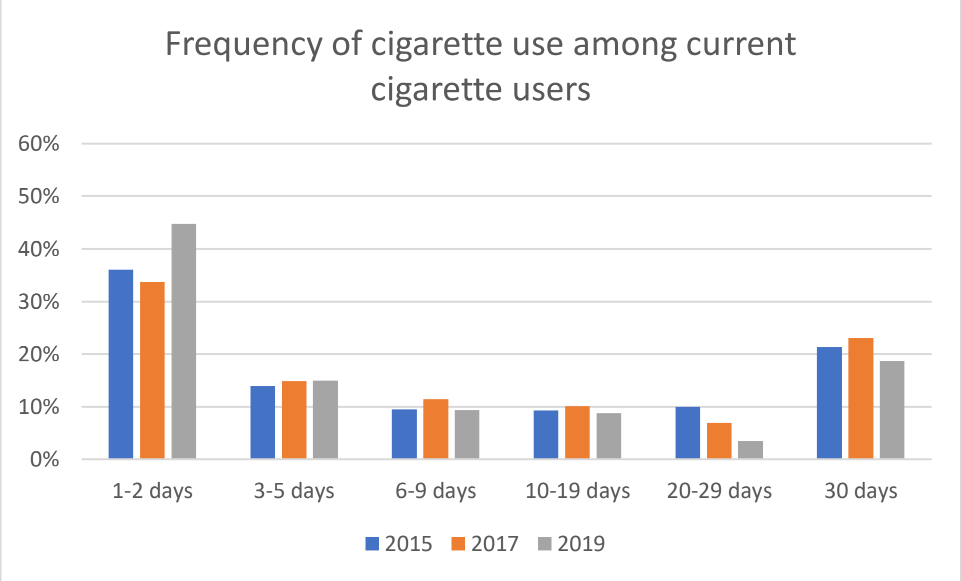

Between 2015 and 2019, e-cigarette days used per month shifted from light use to heavier use in the YRBSS (top panel). The intensities of cigarette consumption generally did not change over time.

© 2022 Glantz S et al. *JAMA Network Open*.

eFigure 5. Percentage of e-Cigarette and Cigarette Users Who Used Product ≥20 d/mo

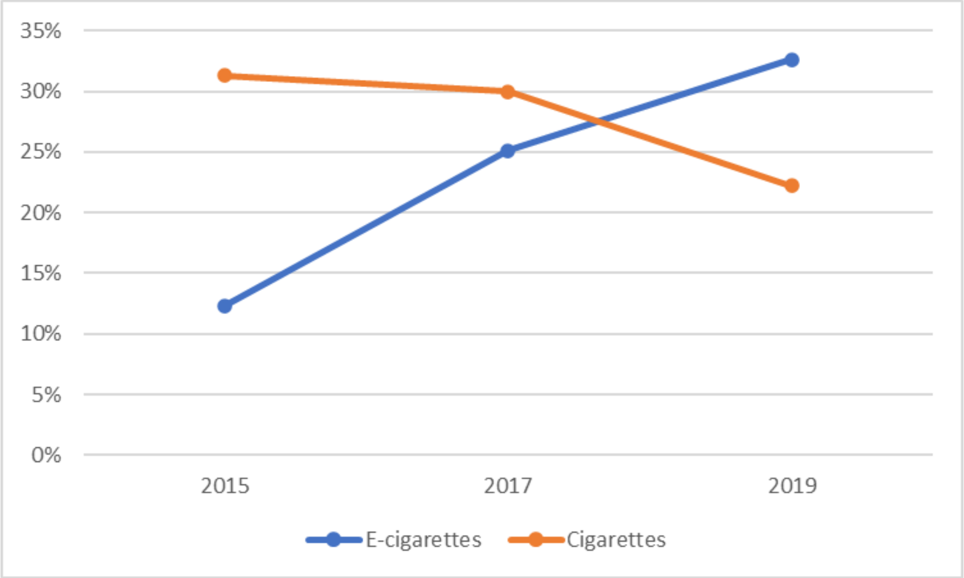

Between 2015 and 2019, the percentage of e-cigarette users that used 20+ days per month shifted from 12% to 33%, while the percentage of cigarette users that used 20+ days per month shifted from 31% to 22% in YRBSS.

© 2022 Glantz S et al. *JAMA Network Open*.
